# Parenting, Child Maltreatment, and Social Disadvantage: A Population-Based Implementation and Evaluation of the Triple P System of Evidence-Based Parenting Support

**DOI:** 10.1177/10775595241259994

**Published:** 2024-06-06

**Authors:** Matthew R. Sanders, Denise Clague, Tomasz Zając, Janeen Baxter, Mark Western, Carys Chainey, Alina Morawska, Wojtek Tomaszewski, Ronald J. Prinz, Kylie Burke

**Affiliations:** 1 Parenting and Family Support Centre, School of Psychology, 1974 The University of Queensland, Brisbane, QLD, Australia; 2 Australian Research Council Centre of Excellence for Children and Families Over the Life Course, The University of Queensland, Brisbane, QLD, Australia; 3 Institute for Social Science Research, 1974 The University of Queensland, Brisbane, QLD, Australia; 4 Research Center for Child Well-Being, 2629 University of South Carolina, Columbia, SC, USA; 5 Metro North Health Service – Mental Health, Brisbane, QLD, Australia

**Keywords:** prevention, child maltreatment, population-based, parenting, evidence-based parenting support

## Abstract

Child Maltreatment (CM) is a widespread public health problem, with adverse outcomes for children, families, and communities. Evidence-based parenting support delivered via a public health approach may be an effective means to prevent CM. The Every Family 2 population trial applied a public health approach to delivering evidence-based parenting support to prevent CM in disadvantaged communities. Using a quasi-experimental design, 64 matched low socioeconomic communities in the Australian states of Queensland and New South Wales received either the full multi-level Triple P system (TPS) of parenting support, or Care as Usual (CAU). Two population indicators of CM, the number of substantiated cases of CM, and the number of notifications of CM to protective services were compared using Welch’s *t*-test to evaluate intervention effectiveness. After two years of intervention, medium to large effect sizes favoring TPS communities were found for substantiations (d = 0.57, *p* < .05) and notifications (d = 1.86, *p* < .001). These findings show the value of the TPS, deployed using a public health approach, in efforts to prevent CM in socially disadvantaged communities. A number of uncontrolled contextual factors are described that may have contributed to some of the differences detected between TPS and CAU communities.

The prevalence of child maltreatment (CM) internationally and within Australia is alarming. Reports from Western nations such as the United States indicate that more than one in three children are the subject of a child protective services investigation and one in eight children experience confirmed maltreatment before they reach adulthood (Wildeman et al., 2014). Within Australia, the recent Australian Child Maltreatment Study (Higgins et al., 2023), a survey of 8503 people aged 16-65+, showed that during childhood, 28.2% of respondents had experienced physical abuse, 25.7% had experienced sexual abuse, 34.6% had experienced emotional abuse, 10.3% had experienced neglect and 43.8% had experienced exposure to domestic violence. CM is of such significance and scale that it has resulted in calls from the World Health Organization (WHO) for an end to all forms of violence against children as part of the United Nation’s Sustainable Development Goals (Target 16.2; WHO, 2022).

Maltreatment results in adverse short- and long-term outcomes for children, families, and communities. It has consistently been associated with an increased risk of mental health disorders, physical health problems, emotional and relational issues, educational and occupational under-achievement and health risk behaviors across the life course (Austin et al., 2020; Carr et al., 2020; WHO, 2022). These effects are compounded by exposure to other childhood adverse experiences such as household dysfunction, substance abuse, mental illness, mother being treated violently, and criminal behavior in the household (Lyu et al., 2022; Rowell & Neal-Barnett, 2022). Growing up in social disadvantage (poverty, underemployment, housing instability, social isolation, living in neighborhoods characterized as disorganized or with high crime) not only compounds the adverse effects of CM across an individual’s life course but also has intergenerational effects that transfer these impacts from one generation to the next (Scott et al., 2016). Families living in poverty are also over-represented in reports to child protective services and are more likely to be referred for neglect involving an inability to feed, clothe or house a child (Fortson et al., 2016). However, targeted services, including statutory child protection services, reach only a small proportion of the population with intervention often occurring late in the trajectory of family dysfunction associated with serious maltreatment (Sanders et al., 2018). The demands on statutory child protection services, and the complications of intersecting systems for responding to young children at risk in the context of family law disputes, increase the need for addressing the primary drivers of maltreatment across the population before the problems become intractable and more difficult to remediate.

Of all the potentially modifiable risk and protective factors that can help to prevent or reduce or mitigate the adverse effects of social disadvantage and CM, evidence-based parenting support is a critical component (Doyle et al., 2023; Sanders et al., 2022; WHO, 2022). Chen and Chan (2016) in their meta-analysis examining the effectiveness of parenting interventions in reducing CM and modifying associated risk and protective factors found that parenting programs successfully reduced substantiated and self-reported CM reports and reduced the potential for CM when implemented as primary, secondary, or tertiary level interventions. Their results also indicated that parenting programs reduced risk factors and enhanced protective factors associated with CM. The authors concluded that parenting programs were an effective public health approach to reduce CM. In Australia’s context, the National Framework for Protecting Australia’s Children 2009–2020 (Council of Australian Governments, 2009) explicitly highlights a move to the adoption of a public health model comprising largely of universal (primary) services such as parenting programs for protecting children from harms associated with CM, noting that while tertiary interventions are critical, it is the primary (universal) services that should comprise the largest component of the service system. Population-based approaches to parenting support aim to address a range of issues, from high-frequency widespread concerns (e.g., less-than-optimal parenting) to relatively common risk factors that drive demand for services/statutory responses, including low-frequency but serious problems (where a blend of universal and targeted strategies may be needed).

While limited in number, studies evaluating the population-based approach to evidence-based parenting support that blend universal components for all families with selected and indicated components for more vulnerable parents have been shown to reduce the prevalence rates of CM and to be cost-effective (Foster et al., 2008; Prinz et al., 2009, 2016; Sanders & Mazzucchelli, 2022). Positive effects have also been demonstrated within socially disadvantaged communities (Mejia et al., 2015; Prinz et al., 2009, 2016). The Triple P – Positive Parenting program is the only fully integrated multi-level, multi-modal, multi-disciplinary intervention that blends universal and targeted parenting support irrespective of individual circumstances or severity of need. The Triple P system (TPS) is not a single intervention. It incorporates five levels of intervention on a tiered continuum of increasing strength and narrowing population reach for parents of children from birth to age 16 (see Table 1 for a more detailed description) thus making it a good exemplar of an evidence-based parenting support delivered within a public health framework.

**Table 1.** Triple P -Positive Parenting Population-Based System of Parenting Support.

Triple P level	Rationale	Description	Public health model alignment
*Level 1 Communications strategy*	Implementation of media and informational strategies on positive parenting.	A localized communication strategy including the use of radio, local newspapers, newsletters at schools, mass mailings to family households, presence at community events, and website information.	Universal (Primary)
	Normalize and destigmatize parenting and family support, make effective parenting strategies readily accessible to all parents, and facilitate and encourage help-seeking and self-regulation by parents who need higher intensity intervention.		
*Level 2 Brief parenting advice.*	Low-intensity programs offering general parenting information and advice as well as on specific concerns about children’s behaviour or development.	Seminar series: 3 independent 90-min sessions designed for delivery to large groups of parents.	Universal/Selected
	Useful for a large proportion of families.	Parents can attend any or all of them and still benefit.	
	Also normalises help-seeking for parenting support.		
*Level 3 Narrow-focused parenting skills training.*	Low to moderate intensity programs.	Brief consultation modality - *Primary Care:* Series of 3 to 4 brief (20-min) consultations (face-to-face or telephone) incorporating active skills training and the selective use of parenting tip sheets covering common developmental and behavioural problems of preadolescent children.	
	Appropriate for management of discrete child problem behaviors not complicated by other major behaviour management difficulties or significant family problems.		
	Children may have mild to moderate behavioural difficulties.		
	Provision of advice and information is supported by active skills training for those parents who require it to implement the recommended parenting strategies.	*Triple P Discussion Groups.* Series of 2-h stand-alone group sessions targeting a specific topic (e.g., disobedience, hassle-free shopping, managing fighting and aggression, developing good bedtime routines).	
	Parents attend the topics that are relevant to them.		
*Level 4 Broad-focused parenting skills training.*	Moderate to high-intensity programs suitable for children who have detectable problems but who may or may not yet meet diagnostic criteria for a behavioral disorder, and parents who are struggling with parenting challenges.	*Standard Triple P* - intensive program delivered as ten 1-h individual sessions;	Selected and indicated
	Parents learn a variety of child management skills, in group, online or individual settings.	*Group Triple P* - attendance at five 2-h group sessions with approximately ten other parents and three brief individually tailored telephone sessions	
	Provision of information plus active skills training and support	*Online Triple P* – 8-module interactive online program guiding parents through Triple P’s core parenting skills.	
	Teaches parents to apply skills to a broad range of target behaviours with their child and siblings.		
*Level 5 – Intensive family intervention: Enhanced Triple P*	High-intensity program offering additional support for families with severe problems	Includes optional intervention modules on partner communication, mood management and stress coping skills for parents, and additional practice sessions addressing parent–child issues.	Indicated
	An optional augmentation of standard (Level 4) Triple P for families with additional risk factors.		
	Families typically complete standard Triple P first.		

The TPS is based on the logic model described by Sanders and Mazzucchelli (2022). The model blends theoretical perspectives derived from social learning theory, self-regulation theory, applied behavior analysis, cognitive behavior principles, developmental theory, and principles derived from the fields of public health, implementation science, and economics to explain change in behavior at the community-wide level. The approach aims to concurrently target modifiable levers to enhance program reach including program design features (in-person, online, brief vs. more intensive), strategies to address parental concerns, enhance motivation to participate, improve emotional well-being, modify dysfunctional cognitions, increase cultural relevance, and increase social support for parents. These mechanisms are hypothesized to produce meaningful change in parenting and ultimately positively influence child outcomes.

Several lines of evidence support the adoption of a population approach to parenting support. First, there are specific studies examining the effects of Triple P on CM. In a landmark randomized place-based experiment in the field of CM, Prinz et al. (2009, 2016) demonstrated that community-wide implementation of the TPS reduced the population prevalence of CM. “Place” in this study was a county with a population between 50,000 and 175,000 people. Randomizing 18 counties in South Carolina to either the intervention or usual services, the US study implemented the full five-level TPS. None of the 18 counties had any prior exposure to Triple P. The nine intervention counties received the Triple P, a tiered, multi-level approach to parenting support, through the existing workforce across several service sectors. Controlling for the five-year baseline period prior to intervention, the study found significant reductions in rates of substantiated CM cases in the statutory child protection service, out-of-home care placements (i.e., foster care), and hospital-treated CM injuries compared with the comparison counties (Prinz et al., 2009, 2016).

Two other quasi-experimental evaluations of the TPS targeted the social and emotional problems of children. Fives et al. (2014) and Doyle et al. (2018) reported on the outcomes of a population-based, quasi-experimental study in Ireland. The implementation of the TPS comprised social marketing, low-intensity parenting seminars (mainly delivered through schools—a valuable hub for non-stigmatizing population-based service delivery), topic-specific workshops on common problems at different developmental stages (e.g., shopping trips, visiting), and an eight-session Group Triple P intervention. The findings showed reduced levels of serious behavioral and emotional problems in children by 37% over a 2.5-year period (Doyle et al., 2018; Fives et al., 2014). Another quasi-experimental evaluation of the TPS known as the *Every Family* population trial was conducted in Queensland, New South Wales and Victoria in Australia (Sanders et al., 2008). Program outcomes were assessed through a computer-assisted telephone interview of a random sample of households (*N* = 3000) in TPS (*N* = 10) and Care as Usual CAU (*N* = 10) communities prior to and again two years post-intervention. At post-intervention, there were significantly greater reductions in the TPS communities compared to CAU communities in the number of children with clinically elevated and borderline behavioral and emotional problems. Parents in TPS communities also reported a greater reduction in the prevalence of depression, stress and coercive parenting.

Collectively these studies show promise that the systemic implementation of the TPS across a community can reduce rates of CM and social and emotional problems in children. However, the field needs to know more about the extent to which positive effects occur when parenting problems are complicated by social disadvantage where families experience additional high levels of stress and intergenerational disadvantage. The present study sought to fill this gap by focusing exclusively on program implementation in relatively disadvantaged areas in Australia.

The RE-AIM framework, developed by Glasgow et al. (1999) was used to guide the implementation and evaluation of the intervention. The essential elements of the RE-AIM framework are (a) Reach—the proportion of the target population that participates in the intervention and also how representative the sample is, (b) Efficacy/Effectiveness—the success rate achieved when the intervention is implemented with fidelity, (c) Adoption—proportion of agencies and settings that adopt the intervention and have staff trained to implement the program, (d) Implementation—the extent to which the intervention is implemented as intended by the adopting agencies, and (e) Maintenance—the extent to which intervention effects are maintained over time and the program is sustained by institutions or agencies.

The context for the present study was the Statewide implementation in Queensland of the TPS. In 2015, the Queensland Government’s Department of Communities, Child Safety and Disabilities funded a statewide roll-out of the TPS, the Queensland Government Triple P Implementation. The goal of this policy-based government initiative was to provide access for all Queensland families to Triple P with an initial target of 140,000 participants statewide between 2015 and 2017. The goal was to implement all five levels of the TPS and variants suitable for families with children aged 2–16 years. The Queensland Government initiative was managed by Triple P International who were responsible for all deliverables.

A place-based study known as *Every Family 2* commenced in August 2017 to run in parallel with the Queensland Government Triple P Implementation. *Every Family 2* aimed to extend the available evidence for population-level effects of the TPS on CM by using Australian administrative data and implementing exclusively within the most socially disadvantaged communities in Queensland and to compare them to closely matched communities in New South Wales using a quasi-experimental design. *Every Family 2* targeted families of children aged 3–8 years, given the critical developmental period of early childhood and the transition to school. Our primary hypotheses were that after three years of implementation, communities exposed to the TPS compared to communities that received Care as Usual (CAU) would have: (1) lower rates of substantiated cases of CM; and (2) lower rates of notifications of CM to child protection authorities.

## Method

### Design

As the current study was conducted in parallel with the government-funded statewide implementation of the TPS it was not possible to adequately isolate communities in order to effectively randomize individual communities to condition and therefore not feasible to employ a randomized controlled experimental design. To achieve the primary goal of the study, to estimate the effect of the TPS on population level indicators of child wellbeing associated with social disadvantage, a quasi-experimental design was implemented using closest match techniques to account for differences between the TPS intervention communities and a set of comparison communities receiving CAU. The implementation phase of the project commenced in August 2017 and continued through to December 2019. This phase consisted of activities associated with the adoption and implementation phases of the Reach, Effectiveness, Adoption, Implementation and Maintenance (RE-AIM) model. This model has been extensively used to evaluate how an intervention interacts with recipients (and service providers) to influence program reach, adoption, implementation, maintenance, and effectiveness. We obtained approval for the project from the research ethics committee at the corresponding authors institution (Approval# HE000941).

#### TPS Intervention Communities

As this trial targeted families in areas of relatively greater disadvantage, the intervention communities were selected from areas with high scores of relative socio-economic disadvantage measured by the Australian Bureau of Statistics (ABS) Socio-Economic Indexes for Areas (SEIFA) indices for the Queensland region using 2011 census data. The ABS geographically defined statistical area level 2 (SA2) represented each community. An SA2 comprises a single suburb or many suburbs that share roads, community facilities, social or historical links, socio-economic characteristics, Local Government Area (LGA) boundaries and a population between 3,000 and 25,000 people (Australian Bureau of Statistics, 2021). However, given that most services, schools and other community infrastructure extends past the SA2 to at minimum the LGA level, we selected the LGA as the Implementation Zone for delivery of the TPS. To focus on disadvantaged communities and to maximise statistical power, we selected Queensland LGAs containing the most SA2s that were: (1) in the bottom 35% of the ABS SEIFA index of relative socio-economic disadvantage and advantage (IRSAD); and (2) had a child population of at least 300 children aged between 3 to 8 years old. Communities with a large proportion of Aboriginal and/or Torres Strait Islander residents (over 30%) were excluded to avoid confounding the results of the present study with another community-wide evaluation of a culturally adapted version of Triple P in a regional indigenous community in Queensland. This process resulted in the selection of three LGAs which consisted of a combined total of 32 Intervention SA2s: (1) Ipswich (*n* = 11); (2) Moreton Bay (*n* = 13); and (3) Toowoomba (*n* = 8). Any parent including indigenous parents could participate in Triple P if they resided in the targeted TPS community.

#### Care as Usual Comparison Communities

CAU communities were established by first generating a pool of SA2s in other Australian states to be matched as control communities. States which had received extensive delivery of the TPS or where there were current state-funded rollouts of the program were first eliminated from the pool. A single state, New South Wales (NSW), was then selected, and matching criteria were defined to identify communities comparable to the intervention (TPS) communities by applying the same eligibility criteria across demographic and location variables. In addition, to be eligible as a comparison community in matching analyses, the community could not have had exposure to the full TPS. It is possible that individual Triple P programs were offered in the comparison communities but not the TPS or other similarly comprehensive Government initiatives during the implementation period.

State-wide matches for each TPS community were identified using ABS 2011 census data, Australian Early Development Census data and Accessibility/Remoteness Index of Australia (ARIA) data. Matching parameters were as follows: Child population of 3 to 8 year olds is +/− 5% children of qualifying SA2 child population; Australian Index of Relative Socio-economic Advantage and Disadvantage (IRSAD) is +/− 5% of qualifying SA2 Australian IRSAD; Indigenous population is +/− 5% of qualifying SA2 indigenous population; ARIA Score, Area and Class match qualifying SA2 ARIA classification; Total SA2 population is +/− 5% of qualifying SA2 range; percentage of low income families is +/− 5% of qualifying SA2 range; percentage of high income families is +/− 5% of qualifying SA2 range; percentage of jobless parents is +/− 5% of qualifying SA2 range; percentage of unemployment is +/− 5% of qualifying SA2 range; percentage of single parents is +/− 5% of qualifying SA2 range; percentage of individuals who did not complete high school is +/− 5% of qualifying SA2 range; percentage of individuals born in Non-English speaking countries is +/− 5% of qualifying SA2 range; percentage of children in the bottom 25% in each Australian Early Development Census domain (health, social, emotional, language and communication) are +/− 5% of qualifying SA2 ranges.

The *caseMatch* package (Nielson, 2016) was used to identify the most similar NSW SA2s for each intervention SA2 based on Euclidean distance calculated for standardized demographic variables and Australian Early Development Census results (z-scores). The ARIA category was also used as a matching variable. All matching variables were weighted equally. In the caseMatch package the number of matches to return can be defined. The limit was set to 79 so all NSW SA2s in the matching pool would be returned in order of similarity to the target intervention SA2 from most similar to least similar. The following rules were applied to obtain a match for each intervention SA2: (1) The highest ranked NSW SA2 based on Euclidean distance was assigned to an intervention SA2 if that NSW SA2 was a unique match; (2) when the highest ranked NSW SA2 was not a unique match, the NSW SA2 was matched to the intervention SA2 that had no closer matches compared to the other matching intervention SA2s based on Euclidean distance; (3) the matched NSW SA2 and intervention SA2 were removed from the pool; and (4) the process above was repeated with the next highest ranked NSW SA2 for each intervention SA2, until all intervention SA2s had one match. Supplemental Table 1 provides a deidentified list of the TPS and CAU matches and associated Euclidean distances.

#### Defining Reach

The project and delivery of Triple P targeted families with at least one child aged 3–8 years old living in areas with relatively greater disadvantage. Based on the 2011 Australian census, there were 350,692 children aged 3–8 years old in Queensland, of which 100,944 (representing a proportion of 28.8%) resided in the most (lowest 35%) disadvantaged areas according to SEIFA 2011. The population of children aged 3–8 years old living in the identified TPS intervention communities was nearly 30,000.

### Every Family 2 Implementation Approach

The TPS model involved partnering with existing service provider networks, local schools and community organizations. Professional training was offered to a multidisciplinary mix of service providers who had the capacity to implement Triple P from various community organizations in the health, education, and welfare sectors. The implementation approach involved a hybrid model in which the capacity to deliver Triple P directly by community organizations and practitioners was built via Queensland Government offered training and combined with a dedicated team of practitioners directly employed by Every Family 2 who supported and supplemented delivery throughout the implementation zones. The partnered organizations and practitioners committed to completion of a set number of Triple P programs in return for free access to training and post-training support from Triple P International. The TPS team was available to these organizations for additional support as required. In addition, the TPS team directly engaged with organizations across the LGAs to deliver programs on behalf of host agencies and/or as community-based events.

### The Triple P – Positive Parenting System

The TPS incorporates five levels of intervention on a tiered continuum of increasing strength and narrowing population reach (see Table 1). The variants for parents of 2- to 12-year-olds were implemented for the TPS intervention. While families with children aged 3–8 years were targeted for this project, families of children outside this age range were not excluded from attending. All programming levels of Triple P have intervention manuals that have been carefully developed, systematic training regimens for providers/practitioners, and coordinated resource material for parents (videos, workbooks, and tip sheets).

### Adoption

#### Agency Engagement

The TPS team joined local networks within each LGA consisting of key services and organizations, local government and other key community groups) to establish relationships and partnerships for the promotion and delivery of Triple P across the communities. These interactions focused on ways to localize the implementation to each target LGA and to identify activities that would increase the engagement of socially disadvantaged parents. The TPS team actively sought and fostered relationships with a diverse range of organizations and community agencies, including schools and early childhood centers, non-government organizations, health services, sporting and religious organizations, local businesses and local or state government. Organizational engagement was operationalized as follows. An organization was considered to have been engaged if they agreed verbally or in writing to participate as a partner organization in the *Every Family 2* project by committing to either having staff trained to implement one or more variants of Triple P, or promoting the availability of TPS to families they work with. Levels of engagement by organizations and groups were divided into five categories: Awareness Raising (e.g., displaying flyers or information), Hosting (e.g., offering a venue and providing administrative support for TPS practitioners to deliver a program), Mentoring (e.g., agencies with trained practitioners seeking support to build confidence to deliver, including co-facilitation), Program Delivery (trained practitioners within the organization delivering Triple P and providing data) and Sponsorship (e.g., the provision of funding or infrastructure support to assist embedding of TPS within the community).

#### Training of Practitioners

The Triple P professional training courses involved 2–5 days of active training (see model in Sanders & Murphy-Brennan, 2010). Each two-part training course involved a maximum of 20 practitioners. Part 1 provided basic skills training that involved active exercises including video modeling of skills, small group practice, and peer and trainer feedback. Comprehensive resources were provided that included a practitioner manual, parent workbooks, and video resources practitioners used in training. Part 2 was the accreditation process that was conducted approximately 2 months after the Part 1 training. Practitioners had to demonstrate core competencies in simulated roleplays involving other practitioners. Following accreditation, practitioners participated in monthly peer support sessions that provided ongoing supervision and support. Practitioners also received implementation support from *Every Family 2* project staff.

#### Recruitment

A multi-method approach was taken to promoting the program to families. The TPS team undertook a diverse and targeted promotional and recruitment strategy within each LGA. While the methods were primarily systematically applied in each region, specific strategies were tailored to the local context. The two primary strategies included targeted social media advertising via paid and organic posts using Facebook and the use of printed promotional flyers. The flyers were distributed to agencies who had agreed to support the initiative, as outlined in the Engagement section as well as through “flyer drops” (community-wide drop-offs and pinning of flyers within restaurants, cafes, local businesses and shopping centres). In addition, traditional media was used with newspaper articles, advertising within family focused magazines and school newsletters. Additional paid advertising included promotion on Shopping Centre Smart Screens and Shopalives and within bathroom/parent rooms and via advertising applied to local buses. Local radio played an important role in promotion with advertising spots, recorded interviews and the establishment of a local parenting advice series. Recruitment was further supported by the statewide promotional activities undertaken by the Queensland Government Triple P Implementation team, including strategies such as online and social media advertising and billboards placed outside the intervention regions. Findings from the Brief Participant survey (*N* = 2541) indicated that promotional activities conducted by schools (*n* = 1128; 44%) and on social media (*n* = 625; 25%) were the most reported methods of hearing about the program for attendees. Other methods included their child’s childcare center (*n* = 182; 7.2%), advertising (*n* = 176; 7%), other professionals (e.g., psychologist, counselor; *n* = 313; 12%), and the Family Court (*n* = 117; 4.6%).

### Measures

#### Brief Participant Survey

Participants attending Triple P completed an anonymous brief survey. Surveys captured parent demographic characteristics (e.g., parent age and gender; number of children, suburb in which they live). Demographic data was used to assist with tracking the number of parents across the implementation local government areas and TPS communities, and the program variant (e.g., group or seminar).

#### RaCYN Survey

The Raising Children in Your Neighbourhood (RaCYN) was a cross-sectional telephone household survey of households across TPS and CAU areas. It was designed to determine whether parenting support information and programs were reaching families and to capture parents’ experiences of parenting and beliefs about support for parents in raising their children in their community. The sample was a convenience sample of parents who volunteered to complete the survey anonymously. The survey was conducted 18 months following the commencement of intervention as a check to verify that there was differential exposure to Triple P in the TPS and CAU communities. The number of respondents in the TPS communities was 647 and CAU communities was 206.

#### Administrative Data

State Government Administrative Data were integrated to create a data set for comparing TPS and CAU communities across CM indicators. The process of data access and merging was managed in collaboration with government departments. The Queensland Government’s former Department of Child Safety, Youth and Women provided access to data pertaining to children who had an involvement in any component of the child protection system, including notifications and substantiations. Notifications were defined as reports where there is a reasonable suspicion that a child is in need of protection and substantiations as a finalized investigation classified as ‘substantiated’. In NSW child protection information was accessed through the NSW Government Department of Communities and Justice. Notifications were defined as reports meeting the risk of significant harm threshold, and substantiations based on finalized field assessments classified as ‘substantiated’. These data were supplemented with population size estimates for each SA2 provided by the ABS.

#### Report Data

To contextualize the TPS and CAU communities within their respective states of Queensland (QLD) and New South Wales (NSW), state-wide notification and substantiation rates were obtained from the Report on Government Services 2021 (Australian Government Productivity Commission, 2021). The state-wide data on the pre-intervention period is represented by the 2012-13, 2013-14, and 2014-15 financial years, and the post-intervention period is represented by the 2018-19 financial year.

### Data Analysis Plan

The administrative data were used to compute two population indicators to provide proxy measures for the prevalence of CM in each community in a given year: notification rate and substantiation rate. The notification rate was obtained by first counting children between 3 and 8 who appeared in at least one maltreatment notification in a given year and then using the ABS data on the local populations to calculate the annual rate per 1000 children in the target age range. The second measure was calculated in a similar manner. The only difference was that only children with substantiated notifications were counted in the first step.

For both indicators, the changes between pre-and post-intervention rates for individual communities were examined. The pre-intervention rate was calculated by averaging for each community the annual rates observed between the financial years 2012-13 and 2014-15, reducing the volatility of the indicators over time. The post-intervention rates were captured in the 2018-19 financial year. The rate of change among TPS and CAU communities on substantiation rates was compared using Welch’s t-test, with the community population used as the unit of inference (as per Hemming & Taljaard, 2023). Effect sizes were calculated as Cohen’s d (Cohen, 1988). State-wide trends for QLD and NSW were then examined to contextualize the observed results.

## Results

### Baseline Equivalence of Triple P System Communities and Care as Usual Communities

Table 2 presents findings on the equivalence of TPS and CAU communities at baseline on various indices of socio-economic disadvantage characteristics. These findings show that TPS and CAU communities were well matched. The only statistically significant differences between them were observed on the Australian Early Development Census language and cognitive skills domain, and not for physical health, social competence, and emotional maturity domains.

**Table 2.** Baseline Comparison of Sociodemographic Variables for Triple P System (TPS) and Care as Usual (CAU) Communities.

Characteristic	TPS	CAU	*t*	*p*
	Mean (SD)	Mean (SD)		
Total population	10,881 (5,108)	12,845 (4,319)	−1.66	.308
Largest population in state as %	37.09 (17.45)	38.88 (13.07)	−0.46	.081
Total child population (aged 3 to 8)	932 (513)	985 (397)	−0.46	.227
Total child (aged 3 to 8) population as %	8.59 (1.66)	7.56 (1.24)	2.81	.198
% Indigenous	3.94 (1.80)	3.94 (1.37)	0.00	.271
% Who don’t speak English well or not at all	1.19 (1.47)	1.09 (1.09)	0.29	.980
% Bottom 35% IRSAD	20.06 (9.74)	20.94 (9.02)	−0.37	.599
% single Parent	21.44 (4.61)	21.06 (4.29)	0.34	.612
% Unemployed	7.78 (2.37)	7.78 (2.00)	0.00	.449
% Low income families	5.69 (1.42)	6.25 (1.50)	−1.54	.937
% High income families	54.22 (5.65)	53.31 (5.87)	0.63	.691
% Jobless parents	6.00 (3.69)	4.97 (2.12)	1.37	.272
% Bottom 25% physical health and wellbeing	32.49 (6.66)	30.76 (7.01)	−1.01	.314
% Bottom 25% social competence	29.39 (7.46)	27.02 (6.19)	−1.38	.172
% Bottom 25% emotional maturity	26.9 (7.12)	24.42 (5.13)	−1.59	.117
% Bottom 25% language and cognitive skills	27.05 (8.58)	17.69 (5.21)	−5.28	.000
% Bottom 25% communication skills and general knowledge	32.11 (8.09)	31.49 (7.87)	−0.32	.754

*Note.* Comparison = Independent samples t-test, degrees of freedom = 62.

### Program Reach

Triple P was delivered in-person and online to families in the TPS communities over the 3-year intervention period. Table 3 shows the number of attendees across the LGAs in which TPS communities were located, based on data from the Brief Participant Survey. The total number of attendees during the implementation period was *N* = 5,802, including 227 attendees who did not complete the Brief Participant Survey. While the program was promoted and targeted to families living within one of the three implementation LGAs, 662 attendees were identified as living outside these regions. Table 3 profiles program attendance, with reach defined as attendees represented as a percentage of families with a 3-8-year-old child. There is a potential overestimation of the number of attendees, as individuals were able to attend multiple programs at diverse locations across the state. After 3 years of intervention, the average reach rate within TPS communities was 13% (range 10.8–19.1%). The number of attendees by program type is also summarised in Table 3. Level 2 Triple P was the most attended, with 51.9% of parents/caregivers in TPS communities having received Level 2 Triple P. Triple P Online was the next most common (28.1%), followed by Level 3 (10.9%) and Level 4 (9.1%).

**Table 3.** Triple P Reach Within Triple P System Communities Within Local Government Areas (LGAs): Attendees Between April 2017 and June 2020.

LGA	Family population		LGA attendees	TPS communities attendees					TPS communities reach (%)
	LGA	TPS communities		Level 2	Level 3	Level 4	Online	Total	
Ipswich	12,849	7000	1497	540 (57.45)	37 (3.94)	93 (9.89)	270 (28.72)	940 (100.00)	13.43
Toowoomba	8611	3604	1230	372 (53.99)	68 (9.87)	105 (15.24)	144 (20.90)	689 (100.00)	19.12
Moreton Bay	30,056	11,220	2183	562 (46.48)	205 (16.96)	59 (4.88)	383 (31.68)	1,209 (100.00)	10.78
Total	51,516	21,824	4910	1,474 (51.94)	310 (10.92)	257 (9.06)	797 (28.08)	2,838 (100.00)	13.00

*Note.* Target population = 18–25% of TPS Communities Family Population. Figures in parentheses = row percentages for TPS Communities intervention attendance types. Reach was computed based on number of attendees divided by number of families in the population.

### Participant Characteristics

Most attendees were female and identified as a mother, with the largest proportion raising one child in the 3-8-year-old age range. Of note, but not unexpectedly given that the programs offered target the 2-12-year-old age range, almost a quarter of attendees indicated that they currently had no children aged 3–8 years. Approximately two-thirds of attendees were attending a Triple P program for the first time (See Table 4).

**Table 4.** Program Attendee Characteristics.

Characteristic	*N* (%)
Total	5149
Role in family	
Mother	3549 (68.9)
Father	931 (18.1)
Step-parent, extended family	256 (4.8)
Other	413 (8.1)
Previous attendance at Triple P	
Yes	539 (33.7)
No, this is my first Triple P program/session	1060 (66.3)
Number of children aged 3–8 years cared for	
0	809 (21.6)
1	1570 (42.0)
2	1075 (28.8)
3	230 (6.2)
4	32 (1.0)
5+	13 (0.3)

### Agency Engagement

**Figure 1.**
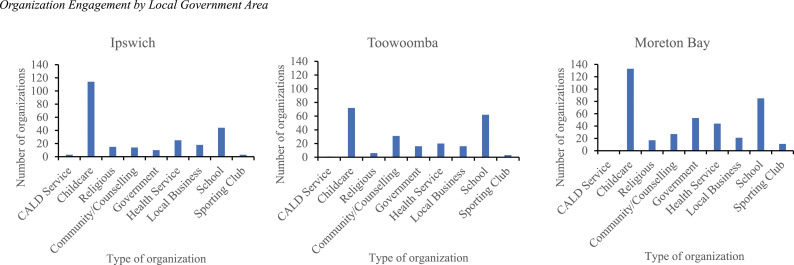
Organization engagement by local government area.

A total of 864 organizations were engaged as part of TPS. Childcare (36.9%) and schools (22.1%) represented the largest proportion of participating organizations, followed by health services (10.3%), government agencies (9.1%), community and counselling organizations (8.3%), local businesses (6.4%), religious organizations (4.4%), sporting clubs (2.0%), and services for culturally and linguistically diverse (CALD) communities (0.5%). Moreton Bay was the largest region and had the most engagements (*n* = 391), while Ipswich (*n* = 246) and Toowoomba (*n* = 227) had similar numbers to one another. Organizations could participate in more than one type of engagement. Across the three LGAs and as would be expected, awareness raising was the most common type of engagement (*n* = 747). Sixty-five distinct organizations hosted programs with most hosting multiple programs. Ten organizations took up Mentoring with another 37 organizations delivering Triple P who agreed to provide TPS data. Five organizations provided Sponsorship, including the provision of office space for TPS local coordinators and funding to support special initiatives such as specifically targeting programs to men. Across the three regions, there was a similar pattern for engagement across agency types (see Figure 1).

The RaCYN survey confirmed that parents from TPS communities had greater awareness of Triple P and were significantly more likely to have participated in a Triple P program compared to parents in CAU communities. A significantly higher proportion of households in the TPS communities (77.9%) had heard of Triple P in the past 12 months than those in CAU communities (43.8%), ꭓ^2^ (1) = 347.83, *p* < .001. A higher proportion of households in the TPS communities (12%, range 10–18%) indicated that they had participated in Triple P compared to CAU communities (6.4%), ꭓ^2^ (1) = 23.41, *p* < .001.

### Comparing Child Maltreatment Between TPS and CAU Communities at Pre-intervention

The TPS and CAU communities were compared with respect to pre-intervention levels for rates of notifications and substantiations (Table 5). These measures were not used to match TPS and CAU. The results show that while there was no statistically significant difference in the substantiation rate between TPS and CAU communities (*p* = .47) before the intervention, the notification rate did differ significantly between the two groups (*p* < .01). TPS communities exhibited a lower notification rate than CAU communities.

**Table 5.** Child Maltreatment-Related Population Outcomes and Percent of Vulnerable Children for Triple P System (TPS) and Care as Usual (CAU) Communities.

	TPS communities		CAU communities		TPS versus CAU on pre-intervention rates	TPS versus CAU on pre-/post-intervention difference	
	Pre-intervention	Post-intervention	Pre-intervention	Post-intervention	*t* (df)	*t* (df)	Effec*t* size
Substantiation rate per 1,000 children	11.61	8.74	10.50	10.86	−0.73 (61.7)	2.27 (61.89)*	0.57
Notification rate per 1,000 children	37.05	33.49	53.24	82.24	N/A	N/A	N/A

*Note.* Effect size: Cohen’s d. Statistical tests of notification rates are not provided due to administrative changes in CAU communities that preclude meaningful comparison. Statistical significance: *p < .05; **p < .01; ***p < .001.

### Effects of TPS on Substantiations and Notifications of Child Maltreatment

Table 5 also presents the mean pre- and post-intervention values of the population outcomes for TPS and CAU communities, along with the results of the statistical tests. For both outcomes, differential trends were found. The notification and substantiation rates dropped on average among TPS communities by 10% and 25%, respectively. In contrast, a reverse trend was observed for CAU communities, (i.e., the notification and substantiation rates increased over time, by 54% and 3%, respectively). The difference between TPS and CAU communities in the rates of change for substantiations was statistically significant, with a medium effect size of *d* = 0.57 at the population level. Significance of the notification rate change difference is not assessed, as the increase observed in CAU communities is likely to be partly attributable to administrative changes outlined in the discussion.

The Report on Government Services data suggest it is unlikely that the observed differences between TPS and CAU communities can be explained by state-wide trends except for notifications in NSW. In Queensland, the state-wide notification rate grew from 18.29 per 1000 children before the intervention to 19.4 per 1000 children after the intervention. In turn, the trend in substantiations showed a decline from 5.9 to 5.2 per 1000 children, which is smaller than that observed for TPS communities. In NSW, the CAU communities followed a state-wide trend in notifications but differed in terms of substantiations. In contrast to CAU communities, the state-wide trend showed a drop in substantiation rates from 9.19 to 8 per 1000 children.

## Discussion

The present study is the first evaluation using administrative data of the effects of a population-based approach to CM prevention in Australia. The results confirm and extend the earlier findings of Prinz et al. (2009, 2016, 2017) that showed the systematic community-wide implementation of the TPS was associated with reduced rates of CM in TPS communities compared to CAU communities. The prior Prinz et al. study showed that Triple P slowed the growth of rates of substantiation. This study extends that work and confirms our primary hypothesis that TPS compared to CAU, was associated with significantly lower rates of both CM substantiations and notifications. Importantly this impact was achieved in socially disadvantaged communities. As socioeconomic disadvantage is a major risk factor for CM (Skinner et al., 2023; Zhang et al., 2022) an intervention that targets all parents in socially disadvantaged communities may somewhat mitigate the adverse effects of poverty on rates of CM. Collectively, these findings demonstrate the feasibility and benefits of using a population-based approach to prevent CM that focuses on promoting the psychosocial well-being of children through effective parenting.

The implementation of multiple levels of TPS took the form of a workforce development strategy that involved training existing multidisciplinary service providers to use evidence-based parenting interventions, supported by a small number of additional project staff providers employed by Every Family 2. The in-service professional training provided was brief and efficient (3 to 6 days depending on program level), utilized a workforce of providers who remained in their existing service roles and service delivery settings, with many additional points of access and engagement for parents created. Prior research has shown that across disciplines, professional training in Triple P is associated with an increase in practitioner self-efficacy (Ma et al., 2023a, 2023b; Sanders et al., 2023). Self-efficacy and organizational support are major predictors of program implementation and sustained program use following training (Ma et al., 2023a; 2023b).

Demand for the program grew gradually over time. The trajectory of attendance growth was similar across all three LGAs. The key form of engagement with communities was partnering with local organizations to generate program awareness. Having programs delivered in convenient nearby community locations was important to facilitate parental engagement. Local early childhood education and care centers and schools played an important role in engaging parents. The pattern of agency engagement was consistent with a public health model–- with the bulk of the delivery comprising universal or selected programs via schools and primary care services while health and community services provided programs for families with higher or more complex needs (selected and indicated). An unknown number of families received the most intensive form of Triple P (Enhanced Triple P, level 5) through community organizations that were not formally affiliated with the TPS initiative. As a result it was not possible to report on the likely small number of families who received level 5 outside through agencies not involved in Every Family 2.

Not all agencies that supported the initiative had the capacity to deliver Triple P programs themselves, and instead hosted and promoted Triple P events, made referrals, and arranged for Every Family 2 project staff to deliver programs. Part of the challenge in a community-wide implementation of a parenting intervention where multiple disciplines, services and agencies are involved, is not knowing how many actual providers exist in the community. While we could track the number of practitioners who were trained in the project to deliver Triple P, there was no master list of parenting practitioners in the target communities, making it nearly impossible to accurately estimate what proportion of eligible practitioners were trained to deliver (or actually delivered) Triple P programs.

Accurately capturing the amount of Triple P intervention taking place in a community proved difficult. This was because community organizations had no obligation or requirement to become part of the Every Family *2* initiative and share their data on implementation. Some organizations or individual practitioners continued to implement Triple P or other programs without being part of the initiative. This led to underestimating the number of families who received Triple P or other parenting interventions. Some organizations in both the TPS and CAU communities were committed to using other parenting programs with a different theoretical orientation and continued to do so during the trial.

The RaCYN household survey confirmed that promotional efforts to increase parental awareness and engagement were successful, and the Every Family 2 initiative achieved participation rates that exceeded the 9% of eligible parents reached in the Prinz et al. (2009, 2016) study. The increased participation rate might be explained by the statewide policy-based implementation of Triple P in Queensland that attracted media attention, the increased use of social media, and access to Triple P online in the current trial. It should be noted that although the program was offered free of charge to all parents, the overall participation rates of parents were still quite modest and below our target rate of 18%. Although we did not achieve our target goal of 18% reach of eligible parents this figure is likely to have underestimated the number of children and parents who benefited from Triple P. Over one-third (36.34%) of participating parents had more than one child (2-5+ children). Parents typically apply their learnings to all relevant children in their family. Parents participating in Triple P are also known to become influencers and provide tips and advice to non-participating parents (friends and family) in their social and extended family networks (Fives et al., 2014). Trained Triple P practitioners frequently report becoming champions and advocate for positive parenting in their workplaces and the wider community contributing to “social contagion” effects (Ma et al., 2023a). When these influences are combined with a strong communications campaign promoting positive parenting the reach of the intervention extends well beyond individual attendance at Triple P sessions.

The present findings need to be interpreted in light of the study’s strengths and limitations. Some noteworthy strengths included the focus on socially disadvantaged communities, the use of the existing workforce to deliver Triple P, the delivery of multiple formats, levels and intensities of the program, and the use of administrative data on CM. In the present study, socially disadvantaged communities were operationalized in terms of SA2 units falling in the bottom 35% within LGAs on the SEIFA index of social disadvantage. By comparison, the prior Prinz et al. (2009) study focused on counties with 21% of children below the U.S. poverty level on average. In both studies practitioners enlisted primarily served socially disadvantaged families.

However, there were also limitations. As we were unable to randomize TPS and CAU communities our capacity to derive causal inferences is reduced somewhat. To ameliorate this limitation, a quasi-experimental approach was used that ensured TPS and CAU communities matched as closely as possible prior to intervention on multiple indices of socioeconomic disadvantage, thus reducing but not eliminating the impact of potential confounding factors. In contrast to the earlier Prinz et al. study, we were unable to report administrative data on rates of out-of-home placements or hospitalization due to CM-related injuries due to different methods of recording such information in QLD and NSW. During the intervention period there were some uncontrollable adverse events, as both states experienced major natural disasters with bushfires/wildfires and floods that affected some of the targeted communities in both states. While it is unknown how these events might have affected rates of CM in the most socially disadvantaged communities, having greater access to evidence-based parenting support during times of environmental crisis is likely to help mitigate the adverse effects of natural disasters on children and parents.

This study also illustrates the complexities of evaluating CM prevention interventions using administrative data from different jurisdictions. Even after careful matching of intervention and comparison communities and evidence that the two groups of communities had differential exposure to the intervention, uncontrolled contextual factors are likely to have contributed towards some of the differences detected between TPS and CAU communities, and also within each state, in rates of substantiations and notifications. For example, state-wide changes were introduced in QLD during the pre-intervention measurement period. In NSW (CAU) there was a 54% increase in notifications observed during the intervention period. Several changes were implemented in this jurisdiction during this period that contributed to this increase including the introduction of a new administrative system that made it easier to add additional children to a Helpline report and the rollout of electronic reporting for all mandatory reporters (Family and Community Services, 2019, 2023). An earlier Child Protection Commission of Inquiry in Queensland (2013) produced several recommendations to address CM in QLD. It is unknown what effects (if any) these recommendations had on rates of CM notification and substantiation. However, when CM rates were compared between TPS communities and other communities in QLD, the reductions in notification and substantiation rates were confined to the TPS communities.

The medium effect size found for CM substantiations is greater than the small effect size reported by Schilling et al. (2020) in North Carolina but smaller than the large effect size (*d* = 1.34) found in the original trial of the Triple P System reported by Prinz (2017). As expected with a universal parenting intervention, most parents participated in a low-intensity level 2 variant of Triple P and Triple P Online. There is increasing evidence that low-intensity variants can produce medium to large effect sizes for both child and parent outcomes. Seminars tend to attract parents who experience significant behavior problems with their children. Recent evidence from Sweden shows that Triple P seminars are associated with medium effect sizes for child outcomes and large effects for parent-reported outcomes (Dahlberg et al., 2022). Also, there is growing evidence that Triple P Online produces comparable effects to Triple P delivered in person (Prinz et al., 2022).

Other unknown factors that may impact these rates include potential changes to staffing, investment, and community awareness of CM. The substantiation measures used in the current study may be less vulnerable to such influences. Finally, as Indigenous children are typically overrepresented in the child protection system, higher rates of program participation of First Nations parents in the TPS communities might have been expected. As this did not occur, additional focused efforts to engage Indigenous parents in culturally adapted variants of Triple P seem warranted (see Andersson et al., 2023). We included both notification and substantiation rates to provide important background contextual information in each state that may affect the interpretation of the findings relating to state-based differences in CM rates.

Despite these limitations and caveats, this study provides an encouraging demonstration that evidence-based parenting support can achieve meaningful population-level impacts on major social problems such as CM in relatively disadvantaged communities. The present findings support the utility of blending the prevention of CM and the promotion of child and family well-being in an integrated public health approach to parenting support (Carmona, 2006). Further research is needed to examine the mechanisms of change explaining population-level change (Sanders & Mazzucchelli, 2022). While considerable evidence shows that participation in Triple P increases parental self-efficacy and reduces parental coercion, there is much to learn about the role of other potential moderators (parental gender, child characteristics, parental adverse childhood experiences, family conflict, and violence) and mediators (changes in parenting practices, parental adjustment, social support, peer to peer advocacy, and collective efficacy).

## Supplemental Material

Supplemental Material - Parenting, Child Maltreatment, and Social Disadvantage: A Population-Based Implementation and Evaluation of the Triple P System of Evidence-Based Parenting Support Supplemental Material for Parenting, Child Maltreatment, and Social Disadvantage: A Population-Based Implementation and Evaluation of the Triple P System of Evidence-Based Parenting Support by Matthew R. Sanders, Denise Clague, Tomasz Zając, Janeen Baxter, Mark Western, Carys Chainey, Alina Morawska, Wojtek Tomaszewski, Ronald J. Prinz, and Kylie Burke in Child Maltreatment.

Supplemental Material - Parenting, Child Maltreatment, and Social Disadvantage: A Population-Based Implementation and Evaluation of the Triple P System of Evidence-Based Parenting Support Supplemental Material for Parenting, Child Maltreatment, and Social Disadvantage: A Population-Based Implementation and Evaluation of the Triple P System of Evidence-Based Parenting Support by Matthew R. Sanders, Denise Clague, Tomasz Zając, Janeen Baxter, Mark Western, Carys Chainey, Alina Morawska, Wojtek Tomaszewski, Ronald J. Prinz, and Kylie Burke in Child Maltreatment.

## Ethical Statement

### Ethical Approval

All procedures performed in studies involving human participants were in accordance with the ethical standards of the institutional research committee and national ethical standards for research, and with the 1964 Helsinki declaration and its later amendments or comparable ethical standards.

## Data Availability Statement

Data associated with this project is owned and stored securely by The University of Queensland, Australia. Requests for access to the data can be made in writing to the corresponding author, Professor Matthew R Sanders.*
