# Supplementary material for: Parenting, Child Maltreatment, and Social Disadvantage: A Population-Based Implementation and Evaluation of the Triple P System of Evidence-Based Parenting Support
Source: Child Maltreat. 2024 Jun 6;30(1):177–91. doi: 10.1177/10775595241259994 (PMC11656618; doi:10.1177/10775595241259994)
Supplement: Supplemental Material - Parenting, Child Maltreatment, and Social Disadvantage: A Population-Based Implementation and Evaluation of the Triple P System of Evidence-Based Parenting Support [file sj-pdf-1-cmx-10.1177_10775595241259994.pdf]

Supplementary Table A.

Deidentified Case Matching Results for Local Government Areas in Queensland (QLD) and New South Wales (NSW)

QLD LGA	NSW Matched Pair No	Euclidean Distance
1	1	6.607319
1	2	10.285482
1	3	10.762149
2	4	4.315165
2	5	5.817322
2	6	15.50876
2	7	7.585276
2	8	21.13306
2	9	10.24426
2	10	46.46644
2	11	8.922169
2	12	14.239599
2	13	21.07128
2	14	14.398043
3	15	6.407042
3	16	6.188972
3	17	13.8851
3	19	11.26061
3	20	18.888952
3	21	13.58319
3	22	12.450302
3	23	4.551536
3	24	10.481145

QLD LGA	NSW Matched Pair No	Euclidean Distance
3	25	6.540733
3	26	13.53272
3	27	5.571689
3	28	11.339875
1	29	12.805452
1	30	8.232433
1	31	14.51531
1	32	7.417775
1	18	8.334087
