# Supplementary material for: Parenting, Child Maltreatment, and Social Disadvantage: A Population-Based Implementation and Evaluation of the Triple P System of Evidence-Based Parenting Support
Source: Child Maltreat. 2024 Jun 6;30(1):177–91. doi: 10.1177/10775595241259994 (PMC11656618; doi:10.1177/10775595241259994)
Supplement: Supplemental Material - Parenting, Child Maltreatment, and Social Disadvantage: A Population-Based Implementation and Evaluation of the Triple P System of Evidence-Based Parenting Support [file sj-pdf-2-cmx-10.1177_10775595241259994.pdf]

Highlights

- Every Family 2 aimed to prevent Child Maltreatment (CM) in disadvantaged areas.
- Triple P system (TPS) was implemented using a public health approach.
- Varied support levels, from low to high intensity, were offered.
- TPS communities showed significant CM reductions.
- A public health approach to parenting may help to prevent CM in disadvantaged communities.
